# Bridging between economy-wide activity and household-level consumption data: Matrices for European countries

**DOI:** 10.1016/j.dib.2020.105395

**Published:** 2020-03-12

**Authors:** Mattia Cai, Toon Vandyck

**Affiliations:** Joint Research Centre, European Commission, 41032 Seville, Spain

**Keywords:** National accounts, Input–Output, Reclassification, Macro models, Micro data, Budget survey, Equity

## Abstract

This dataset represents bridging matrices between two different data classification systems: consumption by purpose (COICOP) and products by activity (CPA). While the former classification is used in household budget and expenditure surveys, the latter represents the industry sector dimension that is typically adopted in national accounts and input–output tables. We collect input data from Eurostat on total household consumption for 35 COICOP and 63 CPA categories for the year 2015. Based on these data, we construct bridging or concordance tables for 30 European countries using recently developed matrix balancing techniques. The resulting tables enable data conversion between consumption- and production-based statistics, facilitating research that integrates macroeconomics, multi-sectoral international trade and heterogeneous agents in household-level expenditure micro-data. Although they are a necessary input in several types of research, they are often constructed on an ad hoc and region-specific basis and not shared publicly. As such, making this dataset available will be useful for computable general equilibrium and input–output models and for carbon footprint and life cycle analyses that incorporate rich consumption micro-data, for instance to shed light on distributional aspects of climate and energy policies. Furthermore, by eliminating a barrier raised by differences in statistical classifications, this dataset may foster collaboration between different research teams and may facilitate soft-linking between complementary analytical tools used for policy support.

Specifications Table

**Table:** 

Subject	Environmental Science (General)
Specific subject area	Distributional analysis; Linking macro modelling and micro data; Carbon footprint analysis; Life cycle analysis; Environmental impacts of consumption; Climate policy; Energy policy
Type of data	Table
How data were acquired	Matrix balancing techniques with Eurostat input data
Data format	Raw
Parameters for data collection	The data is collected from publicly available sources. In particular, we collect data from National Accounts for 30 countries for the year 2015, and fill gaps based on data from proxy years.
Description of data collection	Detailed correspondence tables were collected from the Reference And Management Of Nomenclatures (RAMON) of Eurostat. The data from National Accounts is also collected from Eurostat in both COICOP and CPA classification. We combine these sources to build the dataset with bridging matrices.
Data source location	Institution: European Commission, Joint Research Centre City: Seville Country: Spain
Data accessibility	With the article
Related research article	The methodology used is described in more detail in: Cai, M., & Rueda-Cantuche, J. M. (2019). Bridging macroeconomic data between statistical classifications: the count-seed RAS approach. *Economic Systems Research*, 31(3), 382–403.

**Value of the Data**

• These data are useful for the integration of complementary datasets with different classifications. In particular, these matrices enable a consistent combination of rich micro-data from expenditure surveys with economy-wide statistics from (and models based on) National Accounts.
• These data are particularly valuable for researchers that aim to combine statistics from both the production (national accounts) and the consumption (consumption survey) side in European countries. This includes macro, CGE (computable general equilibrium) and IO (input–output) modelers interested to enrich their framework with disaggregated representation of heterogeneous households, as well as micro-data analysts and modelers looking to add activity-based elements such as (direct and indirect) greenhouse gas emissions embedded in consumption.
• Future work can use these data to shed more light on the carbon footprint across regions [1], cities [2] and, particularly, households [3]. The tables enable an assessment of direct and indirect environmental impact of existing consumption patterns [4], and can be used to extend macro or CGE analyses to cover also distributional impacts of e.g. climate and energy policies [5,6].
• “Data reconciliation between national accounts and other macro data and micro data is a major issue” [7]. The data provided here for 30 countries tackles one barrier for data integration. Sharing these matrices allows other researchers to allocate time and resources to different aspects of data reconciliation, and answers a call voiced in earlier work [8]: “No transformation tables are available, so we have developed these in the study. Diverging principles and inadequate descriptions made this a frustrating task. One conclusion of this exercise is that authoritative linking tables should be made available.”

## 1 Data description

The dataset represents bridging matrices between two classifications: consumption by purpose (COICOP) and product by activity (CPA). It covers 30 countries, including all EU Member States, United Kingdom, Norway and Serbia. The base year is 2015, with proxy years in case of missing numbers in the input data from Eurostat. All values are expressed in monetary terms in million euro, current prices for the year 2015 (or proxy year).

The supplementary file with the dataset contains several worksheets, in the following order:

• *Geo:* explanation of abbreviations for country names.
• *Cpa:* explanation of abbreviations for the classification of products by activity (CPA),
• *Coicop:* explanation of abbreviations for the classification of consumption by purpose,
• *AT-UK:* these 30 worksheets contain the actual bridging matrices between 63 CPA and 35 COICOP categories. The data for each country is covered by a separate worksheet. Values are expressed in million euro of the corresponding year.
• *dataByCOICOP:* this worksheet contains the input data as collected from Eurostat by COICOP classification (nama_10_co3_p3).
• *dataByCPA:* this worksheet contains the input data as collected from Eurostat by CPA classification from the use tables at purchaser prices (naio_10_cp16).
• *Correspondances:* this worksheet contains the input data for the seed matrix (as described below), a detailed qualitative correspondence table as collected from Eurostat.

## 2 Experimental design, materials, and methods

To derive the tables, the input data is fed into the count-seed RAS approach recently developed by Cai and Rueda-Cantuche (2018). We briefly describe the methods here, but for more detail we refer the reader to the work of Cai and Rueda-Cantuche (2018). The dataset in the supplementary file is a direct output of the count-seed RAS method, without further post-processing of transport margins or energy statistics.

The method is designed to estimate an unknown matrix, while row and column totals are known. The approach builds on bi-proportional scaling methods, or what is typically called a RAS procedure in input–output economics. In this field, the RAS approach is routinely applied for matrix balancing. Typically, the procedure starts from an initial guess: the seed or prior matrix. In the count-seed RAS, the prior matrix is constructed by counting the number of items that simultaneously contribute to a given pair of source- and target-classification in a fully disaggregated mapping between two classifications. In our case, this correspondence table comes from Eurostat's metadata centre and matches more than 3000 CPA categories with more than 100 COICOP categories. Using this as a starting point for the matrix structure, the approach then applies bi-proportional scaling methods until convergence is reached when row and column totals match the given input data.

The bridging matrices presented here are entirely based on input data from publicly available sources. Two types of missing data had to be addressed. The first type is the lack of input data for the year 2015 for certain countries. To resolve this issue, we take input data for the year 2014 (Bulgaria and Ireland) and for the year 2011 (Malta). The second type of missing data relates to certain categories that are not available in the input data, for instance due to confidentiality. We overcome this lack of data by calculating or imputing values, in some instances splitting the difference between the total and the missing categories according to shares from proxy countries. These instances have been flagged accordingly in the supplementary data file (in the worksheets containing the input data, *dataByCOICOP* and *dataByCPA*). Furthermore, input data from COICOP and CPA classifications were not consistent for some countries, when the aggregates across categories did not match. Inconsistencies are limited and range within ± 3% for all countries. We address this issue by rescaling the values by CPA categories by country to match COICOP totals.

## Disclaimer

The views expressed are purely those of the authors and may not in any circumstances be regarded as stating an official position of the European Commission.

## Conflict of Interest

The authors declare that they have no known competing financial interests or personal relationships which have, or could be perceived to have, influenced the work reported in this article.

## Appendix Supplementary materials

Image, application 1

Image, application 2
